# CXCL9 and CXCL10 Induce Expression of Nociceptive Ion Channels in Primary Sensory Neurons in Models of HIV-Associated Distal Sensory Polyneuropathy

**DOI:** 10.3390/ijms27010523

**Published:** 2026-01-04

**Authors:** Rebecca Warfield, Jake A. Robinson, Stephen Baak, Rachel M. Podgorski, Tara A. Gabor, Maurizio Caocci, Meng Niu, Andrew D. Miller, Howard S. Fox, Tricia H. Burdo

**Affiliations:** 1 Department of Microbiology, Immunology, and Inflammation, Center for NeuroVirology and Gene Editing, Lewis Katz School of Medicine, Temple University, Philadelphia, PA 19140, USAtag324@drexel.edu (T.A.G.); 2 Department of Medicine, Perelman School of Medicine, University of Pennsylvania, Philadelphia, PA 19104, USA; 3 Department of Medicine, Rutgers Institute of Translational Medicine and Science, Robert Wood Johnson Medical School, Rutgers, The State University of New Jersey, 89 French Street, New Brunswick, NJ 08901, USA; 18baakst@gmail.com (S.B.);; 4 Department of Neurological Sciences, College of Medicine, University of Nebraska Medical Center, Omaha, NE 68198, USA; 5 Department of Population Medicine and Diagnostic Sciences, Section of Anatomic Pathology, Cornell University College of Veterinary Medicine, Ithaca, NY 14853, USA

**Keywords:** HIV, SIV, neuropathy, neuroinflammation

## Abstract

HIV-associated distal sensory polyneuropathy (HIV-DSP) remains prevalent even in the antiretroviral therapy (ART) era. Previously, we identified the upregulation of nociceptive ion channels transient receptor potential vanilloid 1 (TRPV1) and ankyrin 1 (TRPA1) in the dorsal root ganglia (DRG) of simian immunodeficiency virus (SIV)-infected ART-treated macaques. To investigate upstream mechanisms, we performed bulk RNA-seq and pathway analysis on DRGs from uninfected, SIV-infected, and SIV-infected/ART macaques. SIV infection drove strong activation of upstream regulators of interferon γ (IFNγ) and lipopolysaccharide (LPS). Although ART reduced overall IFNγ and LPS pathway activity, the IFNγ-inducible chemokines C-X-C motif chemokine ligand (CXCL)9 and CXCL10 remained significantly upregulated. To determine whether these chemokines influence TRPV1/TRPA1 expression, we treated induced pluripotent stem cell-derived peripheral sensory neurons (iPSC-PSNs) with CXCL9 and CXCL10, which induced a significant increase in TRPV1 but not TRPA1 expression. In parallel experiments, IFNγ but not LPS stimulated monocyte-derived macrophages (MDMs) to release CXCL9 and CXCL10. Conditioned media from IFNγ-treated MDMs modestly increased TRPV1 expression in iPSC-PSNs, and pharmacological inhibition of CXCR3, the receptor of CXCL9/10, did not reduce this effect. Together, these data indicate that persistent IFNγ-driven CXCL9/10 signaling may be one contributor to nociceptor sensitization underlying HIV-DSP, even in the presence of ART.

## 1. Introduction

Neuropathic pain encompasses a wide array of conditions that are classified according to their etiology and the sensitization of the peripheral nervous system (PNS) and the central nervous system (CNS) [1]. Upon peripheral nerve injury, dorsal root ganglion (DRG) neurons initiate a transcriptomic program in response to damage that consists of pro-regenerative, pro-apoptotic, and pro-inflammatory genes. The activation and convergence of these pathways are essential for the recovery of DRG neurons, re-establishment of innervation, and return to homeostatic function [2]. Chronic conditions, such as diabetes, cancer/chemotherapy, and human immunodeficiency virus (HIV), often result in persistent inflammation and both direct and indirect DRG neuron damage [3,4,5,6,7], contributing to neuropathic pain. However, HIV peripheral neuropathy is distinct due to the interplay of unresolved inflammation, pre-antiretroviral therapy (ART) viremic damage, post-ART persistent viral replication, neurotoxic viral proteins, and chronic progression despite treatment [7,8,9]. Identifying underlying mechanisms involved in the HIV-mediated damage to the DRG is integral to furthering the understanding of HIV-associated neuropathic pain.

HIV-associated distal sensory polyneuropathy (HIV-DSP) is the clinical manifestation of HIV-associated neuropathic pain and continues to affect people with HIV (PWH) in the post-ART era [7,8]. Utilizing a simian immunodeficiency virus (SIV)-infected rhesus macaque model to study HIV-DSP, we have demonstrated DRG neuroinflammation, neuronal atrophy, and increased nociceptive ion channel expression in SIV infection and ART [6,10]. Monocyte trafficking to the DRG is associated with the severity of DRG pathology and distal afferent dieback in SIV-infected macaques [6]. With ART initiation, monocyte infiltration into the DRG is lower than in untreated infection, and this reduced monocyte inflammation is associated with nociceptor survival and greater distal afferent fiber densities [10]. As severe DRG pathology was also reduced with ART, potential mechanisms for the persistence of HIV-DSP in the presence of ART were investigated. Outside of direct neuronal insult, exposure of neighboring uninjured nociceptors to residual cytokine drives the development and maintenance of neuropathic pain [11,12,13,14,15,16]. Previously, studies from our group have demonstrated accumulation of CD68+ macrophages in the DRG and elevated expression of nociceptive channels: transient receptor potential vanilloid 1 (TRPV1) and transient receptor potential ankyrin 1 (TRPA1) in the DRG and dorsal horn of SIV-infected, ART-treated animals compared to uninfected controls [17]. Interestingly, no difference in expression of TRPV1 or TRPA1 was observed in SIV-infected ART-naïve macaques [17]. This implies that the mechanism underlying peripheral sensitization in SIV-infected, ART-treated animals is unique from the severe DRG neuron atrophy and immunopathology observed in intreated SIV infection. As pain persists in PWH on ART, determining key transcriptional regulators driving continued PNS sensitization is vital in developing novel therapeutic strategies.

Chronic systemic inflammation is associated with HIV disease progression and increased incidence of HIV-associated comorbidities [18,19]. Following peripheral nerve injury, cytokine exposure, derived from proinflammatory DRG macrophages, can upregulate nociceptive ion channels in DRG neurons and drive peripheral sensitization [11,14,16,20]. Currently, neuroinflammatory changes in the DRG that contribute to peripheral sensitization during SIV infection and treatment with ART remain undetermined. Here, utilizing RNA-seq approaches, we aim to identify alterations in the DRG transcriptome with SIV infection and ART. Additionally, we utilized a human induced pluripotent stem cell-derived peripheral sensory neuron (iPSC-PSN) model to determine the mechanism by which identified mediators influence TRPV1/TRPA1 expression, modeling peripheral sensitization observed in HIV-DSP symptoms.

## 2. Results

### 2.1. IFNγ and LPS Pathways Are Upregulated in the DRG with SIV Infection and Downregulated with ART

**Figure 1:**
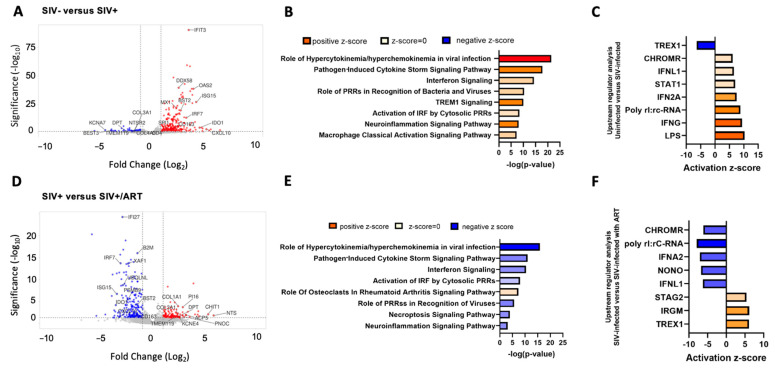
Viral response pathways are upregulated in the DRGs of SIV-infected macaques and decreased in SIV-infected ART-treated macaques. (**A**) Volcano plot of significant differentially expressed genes (DEGs) in the DRGs of SIV- (n = 4) vs. SIV+ macaques (n = 5). Volcano plots indicate the false discovery rate (FDR; −log base 10; cutoff, y = 2 or FDR = 0.01) against fold change (cutoff: x = −2 and x = 2). (**B**) Ingenuity pathway analysis (IPA) of canonical pathway regulation in DRGs of SIV- vs. SIV+ macaques. −log (*p*-value) indicates the confidence of pathway interactions in the DEG network, while the direction of the Z-score indicates upregulation vs. downregulation of the pathway. (**C**) Analysis of upstream regulators of DEGs identified in SIV- vs. SIV+-infected macaques. The strength and direction of the upstream analysis of the DEG network are indicated by the activation Z-score. (**D**) Volcano plot of significant DEGs in the DRGs of SIV+ vs. SIV+/ART (n = 5). Volcano plots indicate the false discovery rate (FDR; −log base 10; cutoff, y = 2 or FDR = 0.01) against fold change (cutoff: x = −2 and x = 2). (**E**) IPA of canonical pathway regulation in DRGs of SIV+ vs. SIV+/ART macaques. −log (*p*-value) indicates the confidence of pathway interactions in the DEG network, while the direction of the Z-score indicates upregulation vs. downregulation of the pathway. (**F**) Analysis of upstream regulators of DEGs identified in SIV+ vs. SIV+/ART macaques. The strength and direction of the upstream analysis of the DEG network are indicated by the activation Z-score. The log_2_ fold change indicates the mean expression level for each gene. Each dot represents one gene. Adjusted *p*-value cutoff of 0.05. LOC694538 currently does not have a published symbol or defined orthologs. Abbreviations: *BEST3*: bestrophin 3, *KCNA7*: potassium voltage-gated channel subfamily A member 7, *TMEM119*: transmembrane protein 119, *DPT*: dermatopontin, *COL3A1*: collagen type III alpha 1 chain, *NTSR2*: neurotensin receptor 2, *COL4A3*: collagen type IV alpha 3 chain, *SPI1*: Spi-1 proto-oncogene, *MX1*: MX Dynamin Like GTPase 1, *BST2*: Bone Marrow Stromal Cell Antigen 2, *DDX58*: retinoic acid-inducible gene I, *IFIT3*: interferon-induced protein with tetratricopeptide repeats 3, *OAS2*: 2′-5′-oligoadenylate synthetase 2, *ISG15*: ISG15 Ubiquitin Like Modifier, *IRF*: Interferon Regulatory Factor 7, *IDO1*: indoleamine 2,3-dioxygenase 1, *CXCL10*: C-X-C motif chemokine ligand 10, *PRRs*: Pattern Recognition Receptors, *TREM1*: Triggering Receptor Expressed On Myeloid Cells 1, *IRF*: Interferon Regulatory Factor, *TREX1*: three prime repair exonuclease 1, *CHROMR*: Cholesterol-Induced Regulator Of Metabolism RNA, *IFNL1*: interferon Lambda 1, *STAT1*: signal transducer and activator of transcription 1, *IFNG*: interferon-gamma, *LPS*: Lipopolysaccharide, *IFI27*: interferon alpha inducible protein 27, *B2M*: beta-2-microglobulin, *XAF1*: XIAP associated factor 1, *UBQLNL*: Ubiquilin-Like Protein, *PSMB9*: proteasome 20S subunit beta 9, *COL1A1*: collagen type I alpha 1 chain, *KCNE4*: potassium voltage-gated channel subfamily E member 4, *PI16*: Peptidase Inhibitor 16, *ACP5*: Acid Phosphatase 5, Tartrate Resistant, *CHIT1*: chitinase 1, *NTS*: neurotensin, *PNOC*: prepronociceptin, *NONO*: non-POU domain containing octamer binding, *STAG2*: Stromal Antigen 2.

RNA-seq of lumbar dorsal root ganglia (LDRG) RNA samples from SIV-, SIV+, and SIV+/ART macaques was performed. The rhesus macaque and SIV genome encompassed 34,608 genes included in the analysis, and transcript abundance was determined as transcripts per million (TPM). A total of 391 genes were significantly differentially expressed between LDRGs from the SIV- and SIV+ animals (Figure 1A). IPA from SIV- to SIV+ comparison showed enrichment of pathways such as hypercytokinemia/hyperchemokinea in viral infection (*CASP1*, *CXCL10*, *IFIT3*, *IL1RN*, *IRF7*, *IRF9*, *ISG15*, *MX1*, *OAS2*), pathogen-induced cytokine storm signaling (*CCL7*, *CD163*, *CGAS*, *CXCL10*, *CXCL11*, *SPI1*, *DHX58*, *TLR1*, *TLR3*, *TLR7*, *TLR8*), and interferon signaling (*CCL7*, *CD86*, *ICAM1*, *JAK2*, *LAT2*, *MYD88*, *NLRC5*) (Figure 1B). IPA identified upstream regulators of LPS, IFNγ, and IFNA2 as drivers in transcriptomic changes identified between SIV- and SIV+ macaque LDRGs (Figure 1C).

Across the comparison of LDRGs from SIV+ and SIV+/ART macaques, 208 genes were significantly differentially expressed (Figure 1D). IPA showed that ART mitigated pathways upregulated by viremic SIV infection conditions. Pathways involving hypercytokinemia/hyperchemokinea in viral infection (*CXCL10*, *IFIT3*, *IRF7*, *ISG15*, *MX1*, *OAS2*), pathogen-induced cytokine storm signaling (*CCL7*, *CCL8*, *CD163*, *DHX58*, *TLR1*, *TLR3*, *CXCL10*, *CXCL11*, *IL21R*), and interferon signaling (*IFI6*, *IFIT1*, *IFIT3*, *ISG15*, *MX1*, *OAS1*, *STAT1*, *STAT2*, *TAP1*) (Figure 1E) were all downregulated in SIV+/ART animals compared to SIV+ animals. Notably, among upregulated genes in SIV+/ART compared to SIV+ macaques were transcripts involved in macrophage immune response (*ACP5*, *TMEM119*, *CHIT1*, *KCNE4*) and pain pathophysiology (*PI16*, *NTS*, *PNOC*) (Figure 1D) [21,22,23,24,25,26,27]. IPA identified IRGM, TREX1, and MAPK1 as upstream regulators of DEGs from SIV+ to SIV+/ART macaques (Figure 1F).

### 2.2. CXCL9 and CXCL10, Downstream Mediators of IFNγ and LPS Signaling, Are Upregulated in the DRGs of SIV+/ART Macaques

**Figure 2:**
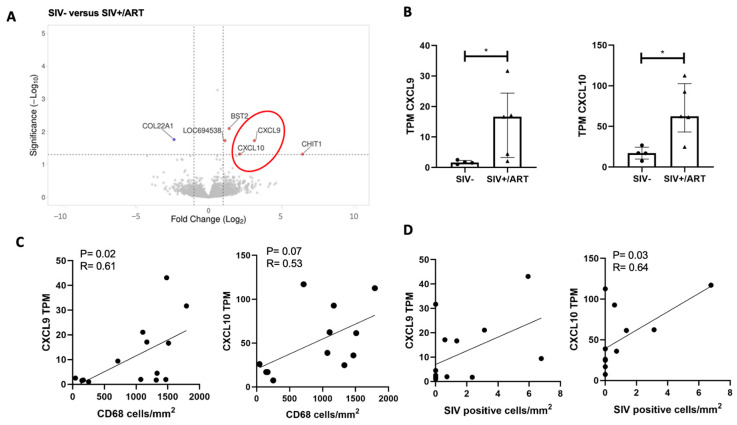
Interferon-inducible chemokines are upregulated in the DRGs of SIV-infected ART-treated macaques and are positively correlated with DRG CD68+ and SIV-RNA+ cell burden. (**A**) Volcano plot of significant DEGs in the DRGs of SIV- (n = 4) vs. SIV+/ART macaques (n = 5). Volcano plots indicate the false discovery rate (FDR; −log base 10; cutoff, y = 2 or FDR = 0.01) against fold change (cutoff: x = −2 and x = 2). The red oval shows the CXCL9 and CXCL10. (**B**) TPM values of *CXCL9* or *CXCL10* in the DRGs of SIV- (n = 4) and SIV+/ART-treated (n = 5) macaques. Statistical analysis was performed using a Mann–Whitney U test * *p* < 0.05. Error bars indicate the median and interquartile range (IQR). (**C**) TPM values of *CXCL9* or *CXCL10* in the DRGs of SIV- and SIV+/ART-treated macaques correlated with the number of CD68 cells/mm^2^ in the DRGs of SIV- (n = 4), SIV+ (n = 5), and SIV+/ART (n = 5) macaques. (**D**) TPM values of *CXCL9* or *CXCL10* in the DRGs of SIV- and SIV+/ART-treated macaques correlated with the number of SIV-RNA+ cells/mm^2^ in the DRGs of SIV- (n = 4), SIV+ (n = 5), and SIV+/ART (n = 5) macaques. (**C**,**D**) Statistical analysis was performed nonparametric Spearman correlation. Error bars indicate the median and interquartile range (IQR). Abbreviations: *CXCL9*: C-X-C motif chemokine ligand 9, *COL22A1*: collagen type XXII alpha 1 chain, *LOC694508*: no ortholog identified.

Six DEGs were identified in the comparison of transcripts from the LDRG of SIV+/ART and SIV- macaques (Figure 2A). *CXCL9* and *COL22A1* were uniquely upregulated in this comparison. Due to limited DEG expression, IPA was not performed. *CXCL9* and *CXCL10* were identified among DEGs and are host response factors to viral infection downstream of IFNγ. There were significant differences both in the CXCL9 TPM (Figure 2B; Mann–Whitney; *p* < 0.05) and CXCL10 TPM (Figure 2B; Mann–Whitney; *p* < 0.05) between SIV- and SIV+/ART macaques. CXCL9 and CXCL10 are type 2 interferon-inducible chemokines secreted by macrophages in response to viral infection [28,29]. CXCL9 TPM were positively correlated with the number of CD68+ macrophages in the DRG for all animals (Figure 2C; Spearman’s rank correlation; *p* = 0.02; R = 0.61), and CXCL10 TPM trended in a positive correlation with CD68+ macrophages in the DRG (*p* = 0.07). In addition, CXCL10 transcripts were positively correlated with the number of SIV RNA+ cells in the DRG of all animals (Figure 2D; Spearman’s rank correlation; *p* = 0.03; R = 0.64). These data show that CXCL9 and CXCL10 are elevated in the SIV model of HIV pathophysiology and HIV-DSP and may play a role in neuropathic pain [30,31].

### 2.3. CXCL9 and CXCL10 Induce TRPV1 and TRPA1 Expression in iPSC-PSNs

**Figure 3:**
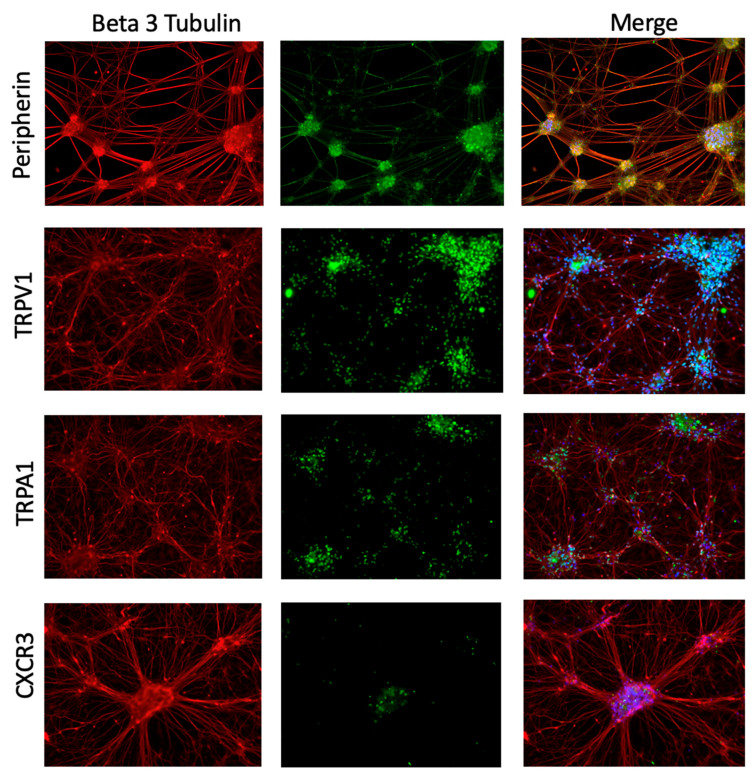
iPSC-PSNs express marker characteristics of peripheral sensory neurons and chemokine receptor CXCR3. Immunocytochemistry of iPSC-PSNs at D40 post-differentiation expresses pan-neuronal marker beta 3 tubulin (all red) and peripheral sensory neuron-specific marker peripherin (green, row 1). At D40 they also express nociceptive ion channels, TRPV1 (green, row 2), and TRPA1 (green, row 3) and chemokine receptor CXCR3 (green, row 4). The overlap of green and red signal (merged) is shown as blue or yellow.

Frozen macaque DRG tissue was available for transcriptomic analyses, but these samples are not viable for in vitro experiments for functional and mechanistic assays. Human iPSC-PSNs provide a scalable and experimentally tractable system of human cells that allowed chemokine-dependent signaling mechanisms relevant to sensory neurons to be tested. iPSC-PSNs were used to model cytokines identified in RNA-seq experiments and their ability to regulate expression of nociceptive ion channels in sensory neurons. The iPSC-PSN protocol is highly characterized [32] to ensure that fully differentiated cells express lineage-specific markers. Confirmatory staining showed iPSC-PSNs express peripheral sensory-specific marker peripherin, TRPV1, TRPA1, and the CXCL9 and CXCL10 receptor CXCR3 (Figure 3).

**Figure 4:**
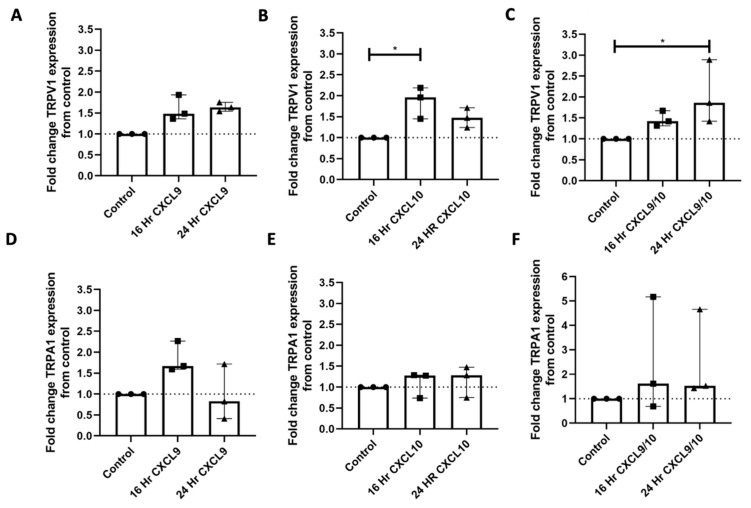
Treatment of iPSC-PSNs with chemokine CXCL10 upregulates TRPV1 expression. (**A**) Fold change *TRPV1* gene expression of IPSC-PSNs (n = 3 independent differentiations) treated with 100 ng/mL CXCL9 for 16 or 24 h (KW: *p* = 0.04). (**B**) Fold change *TRPV1* gene expression of iPSC-PSNs (n = 3 independent differentiations) treated with 100 ng/mL CXCL10 for 16 or 24 h (KW: *p* = 0.03). (**C**) Fold change *TRPV1* gene expression of iPSC-PSNs (n = 3 separate differentiations) treated with 100 ng/mL of CXCL9 and 100 ng/mL CXCL10 for 16 or 24 h (KW: *p* = 0.01). (**D**) Fold change *TRPA1* gene expression of iPSC-PSNs (n = 3 independent differentiations) treated with 100 ng/mL CXCL9 for 16 or 24 h. (**E**) Fold change *TRPA1* gene expression of iPSC-PSNs (n = 3 independent differentiations) treated with 100 ng/mL CXCL10 for 16 or 24 h. (**F**) Fold change *TRPV1* gene expression of iPSC-PSNs (n = 3 independent differentiations) treated with 100 ng/mL of CXCL9 and 100 ng/mL CXCL10 for 16 or 24 h. Each differentiation condition serves as its own control of a fold change baseline of 1. In all graphs, the dotted line represents this control value for comparison. Statistical analysis was performed using a Kruskal–Wallis (KW) one-way analysis of variance followed by a Dunn’s multiple comparison test. * *p* < 0.05. Median and interquartile ranges (IQRs) are shown to accurately reflect the distribution of the data without assuming normality.

To assess the effect of mediators downstream of IFNγ and LPS signaling on *TRPV1* and *TRPA* expression, iPSC-PSNs were treated with CXCL9, CXCL10, or combination CXCL9/CXCL10 for either 16 or 24 h (Figure 4A–F). *TRPV1* expression was significantly higher in iPSC-PSNs treated with CXCL10 for 16 h compared to untreated controls (Figure 4B; Dunn’s multiple comparison, *p* < 0.05). Expression of *TRPV1* was significantly higher in iPSC-PSNs treated with both CXCL9 and CXCL10 for 24 h compared to untreated controls (Figure 4C; Dunn’s multiple comparison, *p* < 0.05). *TRPA1* expression remains unchanged despite treatment with CXCL9 and/or CXCL10 across 16 and 24 h timepoints (Figure 4D–F). For each condition, three independent differentiations of human iPSC-derived peripheral neurons were performed, rather than technical replicates. Because each differentiation was performed on a separate occasion, these biological replicates capture variability inherent to the differentiation process and therefore provide a robust estimate of the phenotype under each condition. Notably, dual treatments with CXCL9 and CXCL10 exhibited high variability, and chemokine levels were not measured during the experiments.

### 2.4. MDMs Increase Secretion of CXCL9 and CXCL10 upon IFNγ Stimulation

**Figure 5:**
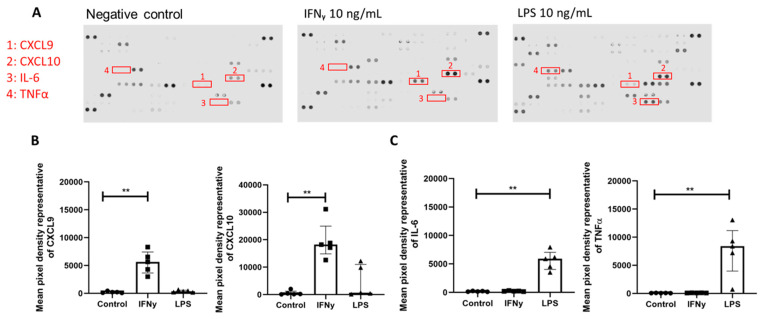
Treatment of monocyte-derived macrophages with upstream mediator IFNγ but not LPS induces enhanced CXCL9 and CXCL10 release. (**A**) Representative images of R&D proteome profiler cytokine arrays. Conditioned media from monocyte-derived macrophages (n = 5) following IFNγ 10 ng/mL or LPS 10 ng/mL treatment at 24 h. Highlighted dots marked 1–4 are representative of CXCL9, CXCL10, IL-6, and TNFα. (**B**) Mean pixel density of dots representing the amounts of CXCL9 (KW: *p* = 0.0009) and CXCL10 (KW: *p* = 0.0004) in conditioned media across treatment groups. (**C**) Mean pixel density of dots representing the amount of IL-6 (KW: *p* = 0.0012) and TNFα (KW: *p* < 0.0001) in conditioned media across treatment groups. Statistical analysis was performed using a Kruskal–Wallis (KW) one-way analysis of variance then a Dunn’s multiple comparison test. ** *p* < 0.01. Error bars indicate the median and IQR.

Since a previous study showed macrophages accumulated in the DRG in SIV+ and SIV+/ART animals, macrophages were exposed to IFNγ or LPS in vitro to identify changes in cytokines to model persistent macrophage activation in the DRG. MDMs were treated with either IFNγ or LPS for 24 h. Global changes in cytokine production were assessed by a profiler array to determine differences in IFNγ or LPS exposure (Figure 5A). The relative CXCL9 and CXCL10 expression was significantly different among treatment groups (Figure 5B; Kruskal–Wallis, *p* = 0.0009 and *p* = 0.0004, respectively), and CXCL9 and CXCL10 were significantly elevated in IFNγ treatment (Dunn’s multiple comparison, *p* < 0.01, *p* < 0.01, respectively). LPS stimulation did not result in increased production of CXCL9 or CXCL10 relative to the control. Proinflammatory cytokines such as IL-6 and TNFα can induce *TRPV1* expression following peripheral nerve injury and are downstream of LPS/TLR4 signaling [14,33]. The relative concentration of IL-6 and TNFα was significantly different among the treatment groups (Figure 5C; Kruskal–Wallis, *p* = 0.0012; Kruskal–Wallis, *p* < 0.0001, respectively), and IL-6 and TNFα were significantly elevated in LPS treatment relative to the unstimulated control (Dunn’s multiple comparison, *p* < 0.01, *p* < 0.01, respectively). IFNγ stimulation did not result in elevated IL-6 or TNFα relative to the control. Confirmatory ELISAs showed that CXCL9 and CXCL10 concentrations significantly differed across MDM treatment conditions (Figure S2B; Kruskal–Wallis, *p* = 0.0006 and Figure S2C; Kruskal–Wallis, *p* < 0.0001, respectively). The concentrations of CXCL9 and CXCL10 were significantly increased following MDM treatment with IFNγ compared to the unstimulated control (Figure S2B; Dunn’s multiple comparison, *p* < 0.01 and Figure S2C; Dunn’s multiple comparison, *p* < 0.01, respectively). LPS exposure did not result in increased CXCL9 or CXCL10 (Figure S2B,C).

In addition, PSNs themselves express the receptors for IFNγ and LPS and can induce expression and release of CXCL9 and CXCL10. iPSC-PSNs were treated with IFNγ and LPS to determine whether direct IFNGR and TLR4 activation can induce increased *TRPV1* expression. Following treatment with IFNγ and LPS, there were no significant differences in iPSC-PSN *TRPV1* expression from untreated controls (Figure S2A). An ELISA performed on media from iPSC-PSNs exposed to IFNγ and LPS identified a significant increase in the release of CXCL10 (Figure S2C, Kruskal–Wallis, *p* = 0.004, Dunn’s multiple comparison, *p* < 0.05) but not CXCL9 (Figure S2B) in neurons treated with IFNγ compared to untreated controls. This indicates that direct stimulation of peripheral sensory neurons with IFNγ or LPS is insufficient to drive CXCL9- and CXCL10-mediated *TRPV1* expression.

### 2.5. IFNγ-Stimulated MDMs Produce Cytokines That Regulate TRPV1 Expression in iPSC-PSNs

**Figure 6:**
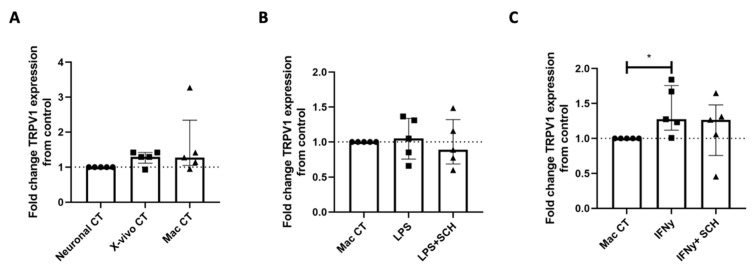
Treatment of iPSC-PSNs with conditioned media from IFNγ but not LPS-treated MDMs can increase *TRPV1* expression. (**A**) Fold change *TRPV1* expression of iPSC-PSNs treated with X-vivo 15 or unconditioned macrophage media as controls. (**B**) Fold change *TRPV1* gene expression of iPSC-PSNs treated with conditioned media from LPS-treated MDMs with and without the addition of CXCR3 inhibitor SCH-546738 (SCH). (**C**) Fold change *TRPV1* gene expression of iPSC-PSNs treated with conditioned media from IFNγ-treated MDMs with and without the addition of CXCR3 inhibitor SCH-546738 (SCH) (KW: *p* = 0.02). Each MDM-unstimulated macrophage control serves as a fold change equal to 1. In all graphs, the dotted line represents this control value for comparison. Statistical analysis was performed using a Kruskal–Wallis one-way analysis of variance followed by a Dunn’s multiple comparison test. * *p* < 0.05. Error bars indicate the median and IQR.

To determine whether *TRPV1* expression is regulated by CXCL9 and CXCL10, iPSC-PSNs were exposed to conditioned media from IFNγ-treated or LPS-treated MDMs in the presence or absence of the CXCR3 antagonist SCH-546738. The iPSC-PSNs treated with macrophage basal media (media control) and media from unstimulated macrophages (conditioned media control) showed no changes in *TRPV1* expression when compared to iPSC-PSNs in basal neuronal media (Figure 6A) verifying that media exposure does not independently regulate *TRPV1* expression. *TRPV1* expression in iPSC-PSNs did not significantly differ following treatment with LPS-stimulated MDM-conditioned media with or without SCH-546738 compared to the unstimulated macrophage control (Figure 6B). This suggests that LPS-induced cytokines IL-6 and TNFα may not directly regulate *TRPV1* expression in iPSC-PSNs. Furthermore, iPSC-PSN expression of *TRPV1* significantly differed when exposed to IFNγ-stimulated MDM-conditioned media with and without SCH-546738 (Figure 6C; Kruskal–Wallis; *p* = 0.02), and while IFNγ-stimulated MDM-conditioned media significantly increased TRPV1 expression (Figure 6C Dunn’s multiple comparison, *p* < 0.05), inhibition of CXCR3 with SCH-546738 did not significantly differ in the post hoc analysis.

## 3. Discussion

To our knowledge, we are the first group to investigate transcriptomic changes in rhesus macaque DRGs during SIV infection and ART. CNS inflammation during SIV/HIV infection is well detailed; however, the neuroinflammatory landscape of the PNS that could contribute to HIV-DSP remains understudied [28,29,34,35]. Previously, we showed upregulation of nociceptive ion channels TRPV1 and TRPA1 in SIV-infected macaques on ART [17]. For the RNA-seq dataset, we did not observe differences in TRPV1 or TRPA1 expression, which may reflect the fact that both genes are low-abundance transcripts that are highly enriched in specific neuronal or immune subpopulations, making them difficult to detect in bulk RNA-seq analyses. In addition, we were unable to examine differences at the cellular level.

RNA-seq identified significant upregulation of genes involving the cytokine response following viral infection. IPA clustered upregulated genes into the viral hypercytokinemia/hyperchemokinea pathway, pathogen-induced cytokine signaling, and the IFN signaling pathway. These findings align with previous studies of transcriptomic changes in the CNS in SIV-infected rhesus macaques, plasma inflammatory profiles of PWH with high plasma viremia, and increased immune cell infiltration to the DRG [6,29,36,37]. IPA identified the two most significant upstream regulators in the DRG during untreated SIV infection as IFNγ and LPS. Microbial translocation in the gut may be a potential source of LPS signaling and has been associated with peripheral leukocyte activation of particularly monocytes in PWH and in SIV-infected rhesus macaques [38,39,40]. Activated monocytes in the periphery respond to chemoattractant molecules such as CCL2 expressed in the DRG and CNS and egress into the nervous system, driving inflammation and accumulation of tissue macrophages [6,35,41,42]. The type 1 and type 2 IFNs are also significant drivers of systemic inflammation during chronic HIV infection [43,44]. IFN signaling is upregulated during HIV and SIV infection in the CNS, lymphoid tissue, and plasma [43,45,46,47,48,49,50]. While activation of IFN signaling is essential for controlling the innate antiviral response, excessive IFN signaling can be maladaptive in perpetuating systemic inflammation by facilitating viral reservoir expansion and CD4+ T cell depletion [51,52,53,54]. Type 1 versus type 2 interferons differ by cognate receptor binding, downstream STAT signaling, and temporal induction following viral infection [53]. In addition, specifically, IFNγ polarizes tissue macrophages to a pro-inflammatory M1 phenotype capable of eliciting neuropathic pain [20,53,55,56].

Transcriptomic changes in the DRGs from SIV-infected to SIV-infected, ART-treated macaques indicate a diminished viral response. We observed a significant downregulation of viral hypercyto/chemokinemia, pathogen-induced cytokine signaling, and IFN signaling pathways. These changes accompanied several log reductions in plasma viral load and a lower number of immune cell infiltrates at necropsy after initiation of ART [10] (Table S1). While high inflammation in untreated animals is largely dampened in the DRG of SIV+/ART, these data show specific residual inflammation in the DRG (Figure 2A). In addition, *CXCL9* and *CXCL10* transcripts are upregulated in the DRGs of SIV-infected, ART-treated macaques compared to uninfected animals, although protein expression was not examined. A caveat of the current study is that the increase in CXCL9/CXCL10 signaling from the macaques is solely based on transcriptomic evidence, so examining CXCL9/CXCL10 at the protein level is an important future direction.

Four of six genes identified as differentially expressed have roles downstream of macrophage polarization and activation favoring the M1 pro-inflammatory phenotype, although here we did not perform immunohistochemistry to confirm their expression in macrophages in NHP DRGs. IFNγ-inducible chemokines *CXCL9* and *CXCL10* are sourced from pro-inflammatory M1 macrophages in models of viral infection [57,58,59]. *CHIT1* encodes the gene for chitotriosidase and is upregulated upon M1 macrophage polarization [58,60]. In addition, we observed downregulation of *COL22A1*, which has implications in M2-mediated wound healing [61]. While we did not detect SIVmac251 viral RNA in the DRGs of SIV-infected or SIV-infected, ART-treated macaques, we did detect upregulation of IFNγ-induced antiviral host restriction factor *BST-2* in the DRGs of SIV-infected, ART-treated macaques. *BST2* works to tether and retain viral particles at the cell surface of HIV-infected macrophages, therefore limiting propagation of infection [62]. This indicates that while proviral replication is low in the DRG, signaling pathways related to viral response are still activated in the DRGs of SIV-infected, ART-treated macaques.

Following peripheral nerve injury, immune cells are recruited to the DRG by a chemotactic axis expressed by glial cells, injured neurons, and activated immune cells [63]. There is a growing body of evidence that chemokines play a role in the initiation and maintenance of neuropathic pain states such as peripheral nerve insult, diabetic nerve damage, and chemotherapy-induced peripheral neuropathy [64]. The family of interferon-inducible chemokines CXCL9, CXCL10, and CXCL11 all bind to their cognate receptor, CXCR3, expressed on sensory neurons. The CXCL9/10/11-CXCR3 chemokine axis plays a role in autoimmunity, cytotoxic T cell recruitment to tumor sites, and viral infection [64,65,66,67,68]. CXCL9 and CXCL10 are significantly increased (~250-fold) in the plasma profiles of PWH compared to pre-infection levels [68]. In addition, plasma concentrations of these chemokines were predictive of HIV disease progression (viral load and CD4 count) and chronic immune activation [68,69]. The combined implications of CXCL9 and CXCL10 in HIV-associated inflammation and *CXCL9/10* upregulation in the DRGs of SIV-infected, ART-treated macaques prompt us to investigate the role of CXCL9/10 in HIV-DSP persistence.

Here, we describe that CXCL9 and CXCL10 can affect the expression of *TRPV1* in iPSC-PSNs in vitro. Specifically, CXCL10 at the 16 h timepoint and dual treatment of CXCL9 and CXCL10 at the 24 h timepoint significantly increase *TRPV1* expression in iPSC-PSNs. CXCL9 and CXCL10 did not lead to any significant changes in the expression of *TRPA1*. In neuropathic pain models, the expression of the *CXCL9* or *CXCL10/CXCR3* axis is increased in the DRG following nerve insult [31,70]. In vitro studies suggest treatment of DRG neurons with CXCL10, but not CXCL9, increases action potential production mediated by activation of p38 and ERK [31]. As *TRPV1/TRPA1* expression and activation are downstream of proinflammatory cytokines/chemokines, ERK, and p38 MAPK signaling, our data suggests that CXCL9/CXCL10 may play a role in HIV-DSP development and maintenance in the ART era [14,71,72,73]. Future studies should determine which downstream signaling mediators drive increased TRPV1 and whether these increases in *TRPV1* expression can directly influence iPSC-PSN sensitization.

As we identified an upregulation of *CXCL9* and *CXCL10* transcripts in RNA-seq, we sought to determine the source of these chemokines using in vitro modeling. CXCL9 and CXCL10 can be produced by a variety of immune cells, glial cells, and peripheral sensory neurons themselves [67]. Utilizing two in vitro models, iPSC-PSNs and MDMs, we assessed the output of CXCL10 and CXCL9 when treated with upstream regulators of DRG gene expression in SIV+/ART macaques, IFNγ or LPS. We determined that iPSC-PSNs treated with IFNγ significantly increased secretion of CXCL10 and produced elevated levels of CXCL9 across treatment groups. Yet there were no significant differences in *TRPV1* expression in neurons treated with IFNγ or LPS compared to untreated controls. This suggests that the amounts of CXCL9 and CXCL10 produced in neurons are insufficient to drive *TRPV1* gene expression in an autocrine fashion. MDMs treated with IFNγ released substantially higher amounts of both CXCL9 and CXCL10 than IPSC-PSNs treated with IFNγ. Thus, we hypothesize macrophages to be capable of producing large amounts of CXCL9 and CXCL10 as an intermediary inducing PSN sensitization.

Following peripheral nerve injury, an influx of macrophages to the DRG is responsible for the initiation and maintenance of neuropathic pain [16,20,74]. Previously, we identified a significantly higher number of CD68+ macrophages in the DRGs of SIV-infected, ART-treated macaques compared to uninfected controls [10]. Innate immune cell macrophages classically produce significant amounts of CXCL10 and CXCL9 following interferon treatment [56,58]. The number of CD68 cells was significantly correlated with an increase in the TPM of CXCL9 and CXCL10 in the DRG. In in vitro experiments, we showed that MDMs treated with IFNγ and LPS elicit specific cytokine profiles. MDMs treated with IFNγ release significantly higher amounts of CXCL9 and CXCL10 relative to unstimulated MDMs, while MDMs treated with LPS do not. In addition, MDMs treated with IFNγ produced significantly more CXCL9 and CXCL10 than iPSC-PSNs treated with IFNγ, which was essentially below the detection limit.

Finally, treatment of iPSC-PSNs with conditioned media from IFNγ-treated MDMs was able to significantly upregulate *TRPV1* gene expression, although at a modest level. Conditioned media from LPS-treated MDMs did not elicit any effect, implying a potential interferon-dependent MDM response in *TRPV1* sensitization of iPSC-PSNs. Blockade of the CXCR3 receptor did not ameliorate the increase in expression of *TRPV1* expression induced by IFNγ-treated MDM-conditioned media, indicating a more complex mechanism than strictly CXCL9 and CXCL10 direct signaling. These findings support a model in which macrophage-neuron crosstalk, rather than direct chemokine receptor activation on neurons, contributes to peripheral sensitization. Moreover, indirect mechanisms involving non-neuronal DRG cell populations, such as satellite glia cells or infiltrating immune cells or a paracrine signaling network with the DRG, may amplify these effects.

Consistent with this model, the number of SIV-RNA+ cells in the DRGs was significantly correlated with CXCL9 and CXCL10 transcript abundance, suggesting that residual viral burden may contribute to sustained chemokine expression despite ART. Although correlative, data suggest a possible link to viral levels and chemokine levels in DRG tissue. HIV/SIV viral proteins are known to exert direct neurotoxic or to indirectly promote neuronal dysfunction through persistent neuroinflammation [75]. Supporting this, conditioned media from HIV-infected macrophages induce axonal retraction and oxidative stress in DRG neurons in vitro [76]. Together, these observations raise the possibility that low-level viral persistence sustains IFN-driven macrophage activation and chemokine production in the DRG, thereby promoting chronic neuronal sensitization. Future studies should define the relative contributions of viral proteins, residual neuroinflammation, and macrophage-derived mediators to CXCL9 and CXCL10 upregulation and sensory neuron dysfunction in the DRGs of SIV+/ART macaques.

## 4. Material and Methods

### 4.1. Animal Model

Uninfected (n = 4) and SIVmac251-infected (n = 10) Indian rhesus macaques (*Macaca mulatta*) were used in this study as a model of HIV infection, as previously described [6,10,36]. Comprehensive descriptions of viral load at necropsy, age, sex, survival post-infection, and DRG pathology for all study animals are included in Supplemental Table S1. Animals A01 to A04 serve as uninfected controls (referred to as SIV-). Animals A05-A14 were infected with SIVmac251 viral swarm (5ng p27; Tulane National Primate Research Center’s Viral Core, Covington, LA, USA) followed by CD8 depletion as previously described (Nonhuman Primate Reagent Resource, Worcester, MA, USA) [6,10,36]. Animals A05–A09 (n = 5) did not receive ART (referred to as SIV+) and were sacrificed upon progression to criteria of humane endpoints for euthanasia. Simian acquired immune deficiency syndrome (AIDS) was diagnosed post-mortem during necropsy for animals in this group [6,10,36]. At 21 days post-infection animals A10–A14 (n = 5) began an ART regimen of raltegravir (22 mg/kg orally twice daily; Merck, Kenilworth, NJ, USA), tenofovir disoproxil fumarate (30 mg/kg subcutaneously once daily; Gilead, Foster City, CA, USA), and emtricitabine (10 mg/kg subcutaneously once daily; Gilead) (referred to as SIV+/ART). SIV+/ART animals were sacrificed at 118 to 120 days post-infection.

### 4.2. Ethics Statement

All tissues used in this study were from historical animals from prior, IACUC-approved studies. All rhesus macaques were maintained at the New England Primate Research center (NEPRC; closed in 2015) or Tulane National Primate Research Center (TNPRC) in accordance with the Association for Assessment and Accreditation of Laboratory Animal Care (AAALAC) standards. All experiments were approved by the NEPRC or the TNRPC’s Animal Care and Use Committees. The TNRPC protocol number is 3497, and the animal welfare assurance number is A4499–01. Euthanasia was consistent with the recommendations of the American Veterinary Medical Association Guidelines for the Euthanasia of Animals [6,10,36].

### 4.3. RNA Isolation from Lumbar DRG Tissue and iPSC-PSNs

Dorsal root ganglia from the lumbar region (L3–L6) were dissected and flash frozen at necropsy using a standard operating procedure and performed by a board-certified veterinarian. Historical flash frozen lumbar DRGs (LDRG, 1–2 per lysate) from each macaque were homogenized in BeadBug^TM^ zirconium prefilled bead tubes (MilliporeSigma, Burlington, MA, USA) with TRIzol (Invitrogen, Waltham, MA, USA) using a BeadBlaster 24 (Benchmark scientific, Sayreville, NJ, USA). Following homogenization, samples were processed for RNA isolation according to the manufacturer’s protocol. A Direct-zol RNA miniprep kit (Zymo Research, Irvine, CA, USA) was used for RNA clean-up, following the manufacturer’s protocol. For RNA isolation from in vitro experiments, iPSC-PSNs were collected, pelleted, and lysed in Monarch RNA lysis buffer, and RNA was isolated with a Monarch total RNA miniprep kit (New England Biolabs, Ipswich, MA, USA). The RNA integrity numbers for samples used were all above 5.

### 4.4. Library Preparation and Sequencing

Library preparation, sequencing, RNA-seq data analysis, and statistical testing were performed by Azenta Life Sciences. RNA samples were quantified using a Qubit 2.0 Fluorometer (ThermoFisher Scientific, Waltham, MA, USA), and RNA integrity was checked with a 4200 TapeStation (Agilent Technologies, Palo Alto, CA, USA). The rRNA depletion sequencing library was prepared by using a QIAGEN FastSelect rRNA HMR Kit (Qiagen, Hilden, Germany). RNA sequencing library preparation was carried out with a NEBNext Ultra II RNA Library Preparation Kit for Illumina following the manufacturer’s recommendations (New England Biolabs, Ipswich, MA, USA). Briefly, enriched RNAs were fragmented for 15 min at 94 °C. First-strand and second strand cDNA were subsequently synthesized. cDNA fragments were end-repaired and adenylated at 3’ends, and universal adapters were ligated to cDNA fragments, followed by index addition and library enrichment with limited cycle PCR. Sequencing libraries were validated using the Agilent Tapestation 4200 (Agilent Technologies, Palo Alto, CA, USA) and quantified using the Qubit 2.0 Fluorometer (ThermoFisher Scientific, Waltham, MA, USA) as well as by quantitative PCR (KAPA Biosystems, Wilmington, MA, USA).

The sequencing libraries were multiplexed and clustered on one flowcell lane. After clustering, the flowcell was loaded on the Illumina HiSeq instrument according to the manufacturer’s instructions. The samples were sequenced using a 2 × 150 Pair-End (PE) configuration. Raw sequence data (.bcl files) generated from Illumina HiSeq was converted into fastq files and demultiplexed using Illumina bcl2fastq program version 2.20. One mismatch was allowed for index sequence identification.

### 4.5. RNA-Seq Data Analysis

After demultiplexing, sequence data was checked for overall quality and yield. Then, raw sequence reads were trimmed to remove possible adapter sequences and nucleotides with poor quality using Trimmomatic v.0.36. The reads were then mapped to the rhesus macaque reference genome (Mmul_10) and the SIV genome (National Center for Biotechnology Information accession no. M33262.1) available on ENSEMBL using the STAR aligner v.2.5.2b. Unique gene hit counts were calculated by using the feature Counts from the Subread package v.1.5.2. Only unique reads that fell within exon regions were counted. After extraction of gene hit counts, the gene hit counts table was used for downstream differential expression analysis, and the subsequent pathway analysis was performed using QIAGEN Ingenuity Pathway Analysis (IPA).

### 4.6. RNAscope

DRG tissues were prepared and sectioned as previously reported [10]. Tissue sections were placed and heated in a 1× target retrieval solution (Advanced Cell Diagnostics, Newark, CA, USA) and treated with a protease plus and a hydrogen peroxide blocker according to the manufacturer’s protocol. SIVmac239 RNAscope probes (homologous sequence to SIVmac251) (Advanced Cell Diagnostics) were hybridized at 40 °C in the HybEZ II Hybridization System. The RNAscope 2.5 HD Assay sequential amplification steps were applied according to the manufacturer’s protocol. Target RNA was visualized through the addition of chromogenic Fast Red A and Fast Red B (Advanced Cell Diagnostics), and sections were counterstained with hematoxylin (Sigma-Aldrich, St. Louis, MO, USA) and mounted using Vectamount (Vector Laboratories, Newark, CA, USA). Tissue sections were imaged using the Keyence BZ-X700 microscope in brightfield for the development of Fast Red chromogen. Images (10–20, 40×) were taken to determine the number of SIVmac251 RNA+ cells per mm^2^ DRG area. SIVmac251 RNA+ cells were counted and divided by the image area (0.0984 mm^2^) to determine cells/mm^2^ where the data are shown as the average value per animal.

### 4.7. iPSC-Derived Peripheral Sensory Neurons

The generation of peripheral sensory neurons was adapted from the following established protocol [32]. iPSC line CHOP WT17.1 (obtained from the Children’s Hospital of Philadelphia (CHOP) Pluripotent Stem Cell Core) [77] was plated on hESC-grade Matrigel maintained in MTesR plus media (Stem Cell Technologies, Cambridge, MA, USA) until 60% confluency. iPSC-PSN differentiation Day 0 (D0) was induced using 100% KSR media (components: 80% KO-DMEM F-12, 20% KO serum replacement, 1× Glutamax, 1× non-essential amino acids, and 10 μM b-mercaptoethanol), which was supplemented with 10 μM SB431542 (Sigma-Aldrich) and 500 nM LDN-193189 (Sigma-Aldrich). From day 2 (D2) to D4, differentiation was continued using the same D0 supplemented 100% KSR media with the addition of 3 μM CHIR-99021 (Stem Cell Technologies), 10 μM DAPT (Sigma-Aldrich), and 10 μM SU-5402 (Sigma-Aldrich). Starting on D4, 25% of the KSR media was replaced every other day with N2 media (comprising 50% DMEM F-12 (Gibco, Waltham, MA, USA), 50% Neurobasal (Gibco), N2 Supplement (Gibco), and B27 Supplement (Gibco)) until the media was fully transitioned to 100% N2 by D10. Starting on D6, SB431542 and LDN-193189 were removed, and the media was supplemented with CHIR-99021, DAPT, and SU-5402.

On D12, cells were replated onto plates coated with poly-ornithine, human plasma fibronectin, and laminin. These cells were then maintained in 100% N2 media supplemented with 10 ng/mL NT3 (R&D Systems, Minneapolis, MN, USA), 10 ng/mL BDNF (R&D Systems), 10 ng/mL NGF (Peprotech, Waltham, MA, USA), and 10 ng/mL GDNF (Peprotech) until D39. On D15, cells were treated for 2 h using D12 media with the addition of 2.5 μg/mL mitomycin C to eliminate dividing cells and purify the culture, after which D12 media without mitomycin C was added. A full media change using D12 media was performed on D18, after which half media changes using D12 media were performed every 5 days until D39. On D39, a half media change was performed using N2 media without neurotrophin supplementation. On D41, iPSC-derived PSNs were fully mature and ready for experimental treatments. A diagram of the iPSC-PSN differentiation timeline is provided in Figure S1. For cytokine studies, D41 iPSC-derived PSNs were treated with either 10 ng/mL IFNγ or 10 ng/mL LPS for 24 h. After 24 h, cells were lysed for RNA extraction, and the conditioned media was collected to measure CXCL9 and CXCL10 concentrations.

### 4.8. Immunocytochemistry

At D12, iPSC-PSNs were plated onto glass bottom dishes coated with poly-ornithine/human plasma fibronectin/laminin. Cells were cultured until D41 as mentioned above and fixed with 4% paraformaldehyde (Electron Microscopy Sciences). Cells were permeabilized with PBST with 0.1% Triton-X100 (Sigma-Aldrich) and blocked using PBST with 2% BSA (Thermofisher) and 5% FBS (Gibco). All antibodies used are detailed in Table 1. Primary antibodies were incubated overnight at 4 °C. We used fluorescently labeled secondary antibodies: Alexa Fluor 488 for detecting anti-rabbit antibodies and Alexa Fluor 647 for detecting anti-mouse antibodies (1:1000) (Thermofisher). Representative 40× images were taken using a Keyence BZ-X700 microscope.

### 4.9. Monocyte-Derived Macrophage (MDM) Culture and Treatments

Human peripheral blood mononuclear cells (PBMCs) from 3 healthy donors were plated at approximately 1 × 10^6^ cells/mL in 6 well plates (n = 3). PBMCs were stimulated with 50 ng/mL recombinant human macrophage colony-stimulating factor (M-CSF) (Peprotech) in X-VIVO15 media (Lonza, Basel, Switzerland) to differentiate monocytes to macrophages. MDMs were differentiated for 7 days and subsequently treated with 10 ng/mL LPS (Thermofisher) or 10 ng/mL IFNγ (R&D Systems) for 24 h. Following treatment, conditioned media was collected for XL cytokine arrays and treatment of iPSC-PSNs.

### 4.10. Proteome Profiler Human XL Cytokine Array Kit

Conditioned media from untreated, IFNγ-treated, and LPS-treated MDMs were probed for cytokines by the Proteome Profiler Human XL Cytokine Array Kit (R&D Systems) according to the manufacturer’s instructions. Following the application of the detection antibody cocktail, Streptavidin-HRP was substituted for IRDye 800CW Streptavidin (1:2000) (Li-Cor, Lincoln, NE, USA) and imaged using an Odyssey CLx imaging system. The signal intensity for each dot representing a cytokine was determined using Image Studio v5.2. Signal intensity from duplicates was averaged and compared between conditions.

### 4.11. Enzyme-Linked Immunosorbent Assay (ELISA)

The concentration of CXCL9 or CXCL10 in conditioned media from iPSC-PSNs or MDMs was determined using a CXCL9 or CXCL10 Quantikine ELISA Kit (R&D Systems). For iPSC-PSN samples, media was spiked with CXCL9 (508.60 pg/mL) or CXCL10 (89.05 pg/mL) to bring media within range of the standard curve. The spike concentration was selected based on the recovery range of 85–95% of the fourth standard for the CXCL9 or CXCL10 ELISAs. The spike amount was subtracted from data to determine the final sample concentration. MDM-conditioned media samples were run without spiking.

### 4.12. CXCR3 Antagonism in MDM Cultures

iPSC-PSNs were treated with conditioned media from untreated, IFNγ-treated, and LPS-treated MDMs from five healthy donors (n = 5). Exposure of iPSC-PSNs was performed by half media change with neuronal media (neuronal control), X-vivo 15 (X-vivo control), untreated MDM media (MDM control), IFNγ-treated MDM media, and LPS-treated MDM media and subsequently incubated for 24 h. CXCR3 antagonism was performed by preincubation of iPSC-PSNs with 12 µM SCH546738 for 15 min (MedChemExpress, Monmouth Junction, NJ, USA) prior to conditioned media treatment. The 12 µM SCH546738 was supplemented with half media changes to maintain the concentration during treatment.

### 4.13. RT-qPCR of iPSC-PSNs

A total of 50 ng of sample RNA per reaction was used with a Luna^®^ Universal Probe One-Step RT-qPCR Kit (New England BioLabs, Ipswich, MA, USA). Reverse transcription and PCR were performed using a Roche Light cycler 96 according to the Luna Universal Probe One-Step RT-qPCR Kit protocol. Primer probe sequences for *TRPV1*, *TRPA1*, and *Beta-actin* (Integrated DNA Technologies, Coralville, IA, USA) are detailed in Table 2. All qPCRs were run in duplicate, and C_T_ values were determined by relative quantification in Lightcycler 96 software. C_T_ values of *TRPV1* and *TRPA1* were normalized to *ACTB* and transformed to fold change in gene expression relative to the control. Fold change from each separate experiment was graphed for a total of n = 3.

### 4.14. Statistical Analysis

Using DESeq2, a comparison of gene expression between the groups of samples was performed [78]. Log2 fold change was calculated, and the *p*-values were generated using the Wald test. Genes with adjusted *p*-values < 0.05 and absolute log2 fold changes > 1 were considered significantly differentially expressed in each comparison. All transcriptomic data will be available upon publication.

For in vitro experiments, all statistical analysis was performed using GraphPad Prism V8. All experimental datasets were tested for normality using an Anderson–Darling test (*p* < 0.05) and assumed to be non-Gaussian in distribution. All comparisons that yielded a *p*-value of *p* ≤ 0.05 (confidence level of 95%) were considered significant. The Mann–Whitney U test was used for comparison between two unpaired measures. The Kruskal–Wallis test for non-parametric one-way analysis of variance was used for comparisons between 3 or more unpaired groups. If found significant, a post hoc Dunn’s multiple comparison test was performed to identify significant differences within the three groups. The results are graphically represented in bar graphs with the median and interquartile range (IQR), displaying individual data points as well. Linear regression analysis was performed to compare co-variance of two variables and statistically compared using Spearman correlations to report the *p*-value and coefficient of determination (R^2^). The results are graphically represented in scatter plots with linear regression mapped to trends.

## Figures and Tables

**Table 1:** Antibodies used in histologic analysis.

Target	Clone	Company	Catalog Number	Dilution
CD68	Mouse (KP1)	Dako, Glostrup, Denmark	M0814	1:400
Beta 3 tubulin	Mouse	Invitrogen, Carlsbad, CA, USA	MA1-118	1:100
Peripherin	Rabbit	Abcam, Cambridge, UK	ab4666	1:1000
TRPV1	Rabbit	Novus Biologicals, Centennial, CO, USA	NBP1-71774	1:100
TRPA1	Rabbit	Novus Biologicals, Centennial, CO, USA	NB110-40763	1:200
CXCR3	Rabbit	Invitrogen, Carlsbad, CA, USA	PA5-23104	1:100

**Table 2:** qPCR primer and probes.

Accession Number	Target Gene	Probe	FWD Primer	REV Primer
NM_080706	*TRPV1*	5′-/56-FAM/TCAAGCAGA/ZEN/GTTTCAGGCAGACACTG/3IABkFQ/-3′	5′-GGCATCATCAACGAAGACC-3′	5′-GGGACCAGGGCAAAGTTC-3′
NM_007332	*TRPA1*	5′-/56-FAM/TGAAGTTCC/ZEN/ACCTGCATAGCTATCCTCT/3IABkFQ/-3′	5′-GACATTGCTGAGGTCCAGAA-3′	5′-GAAACCAAAGTGGCAGCTTC-3′
NM_001101	*Beta-Actin*	5′-/5HEX/AGTTTCGTG/ZEN/GATGCCACAGGACTC/3IABkFQ/-3′	5′-CGTACAGGTCTTTACGGATGTC-3′	5′-GCTCTCTTCCAACCTTCCTTC-3′

## Data Availability

The original contributions presented in this study are included in the article/Supplementary Material. Further inquiries can be directed to the corresponding author(s).

## Supplementary Materials

The following supporting information can be downloaded at: https://www.mdpi.com/article/10.3390/ijms27010523/s1.
