# Supplementary material for: CXCL9 and CXCL10 Induce Expression of Nociceptive Ion Channels in Primary Sensory Neurons in Models of HIV-Associated Distal Sensory Polyneuropathy
Source: Int J Mol Sci. 2026 Jan 4;27(1):523. doi: 10.3390/ijms27010523 (PMC12786925; doi:10.3390/ijms27010523)
Supplement: Supplementary file 1 [file ijms-27-00523-s001.zip › ijms-4026715-supplementary.pdf]

Supplemental Material

CXCL9 and CXCL10 induce expression of nociceptive ion channels in primary sensory neurons in models of HIV-associated distal sensory polyneuropathy

Rebecca Warfield [1], Jake A. Robinson [2], Stephen Baak [3], Rachel M. Podgorski [1], Tara A. Gabor [1], Maurizio Caocci [3], Meng Niu [4], Andrew D. Miller [5], Howard S. Fox [4] and Tricia H. Burdo [3]*

[1]Department of Microbiology, Immunology, and Inflammation, Center for NeuroVirology and Gene Editing, Temple University, Lewis Katz School of Medicine, Philadelphia, PA, USA.
[2]Department of Medicine, Perelman School of Medicine, University of Pennsylvania, Philadelphia PA, USA.
[3]Department of Medicine, Institute of Translational Medicine and Science, Robert Wood Johnson Medical School, Rutgers, The State University of New Jersey, New Brunswick, NJ, USA.
[4]Department of Neurological Sciences, College of Medicine, University of Nebraska Medical Center, Omaha, NE, USA
[5]Department of Population Medicine and Diagnostic Sciences, Section of Anatomic Pathology, Cornell University College of Veterinary Medicine, Ithaca, NY, USA.
*Correspondence: author: Tricia H. Burdo, Ph.D. Henry Rutgers Endowed Professor of Translational Medicine and Science Associate Director, Rutgers Institute for Translational Medicine & Science (RITMS) Rutgers, The State University of New Jersey 89 French Street, Room 4277 New Brunswick, NJ 08901 email: tb874@rbhs.rutgers.edu.

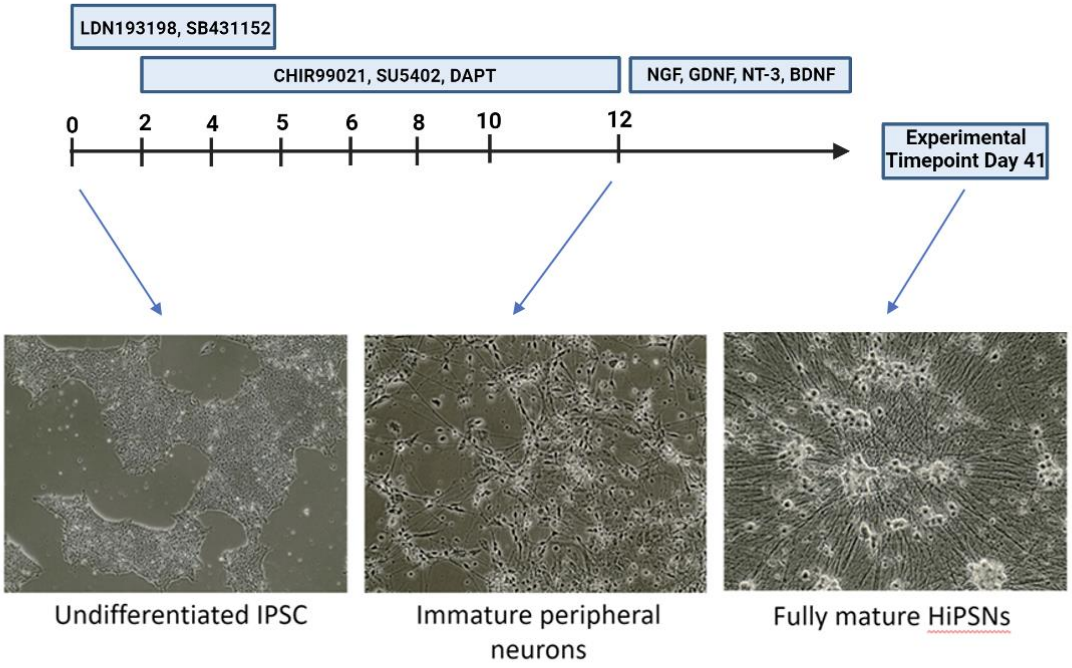

Figure S1. Differentiation timeline for induced pluripotent stem cell-derived peripheral sensory neuron (iPSC-PSN) with morphological changes iPSC line CHOP WT17.1 (obtained from the Children's Hospital of Philadelphia (CHOP) Pluripotent Stem Cell Core) was plated on hESC grade Matrigel maintained in MTesR. iPSC-PSN differentiation Day 0 (D0) was induced using 100% KSR media supplemented with 10 μM SB431542 (Sigma-Aldrich) and 500 nM LDN-193189 (Sigma-Aldrich). On D2-D5 differentiation continues with the 100% KSR media supplemented with SB431542 and LDN-193189 with the addition of: 3 μM CHIR-99021 (Stem cell technologies), 10 μM DAPT (Sigma-Aldrich), and 10 μM SU-5402 (Sigma-Aldrich). On D4, N2 media was added in increasing 25% increments every

other day starting on day 4 until 100% N2 starting on D10. D6-D11 SB431542 and LDN-193189 are removed, and media is supplemented with CHIR-99021, DAPT, and SU-5402. Beginning on D12 of differentiation cells immature iPSC-PSNs were maintained in 100% N2 media supplemented with 10 ng/mL NT3 (R&D systems, Minneapolis, MN) 10 ng/mL BDNF (R&D systems), 10 ng/mL NGF (Peprotech, Waltham, MA), 10 ng/mL GDNF (Peprotech) until D39. On D15 cells are treated with 2.5 μg/mL mitomycin C to eliminate all eliminate dividing cells and purify the culture. Neurotrophin supplement is withdrawn starting on day 39 and treatment of cells occurs on D41 post differentiation. Representative images on day 0 show undifferentiated iPSCs, day 12 images display immature peripheral neurons, and day 41 images illustrate fully mature iPSC-PSNs.

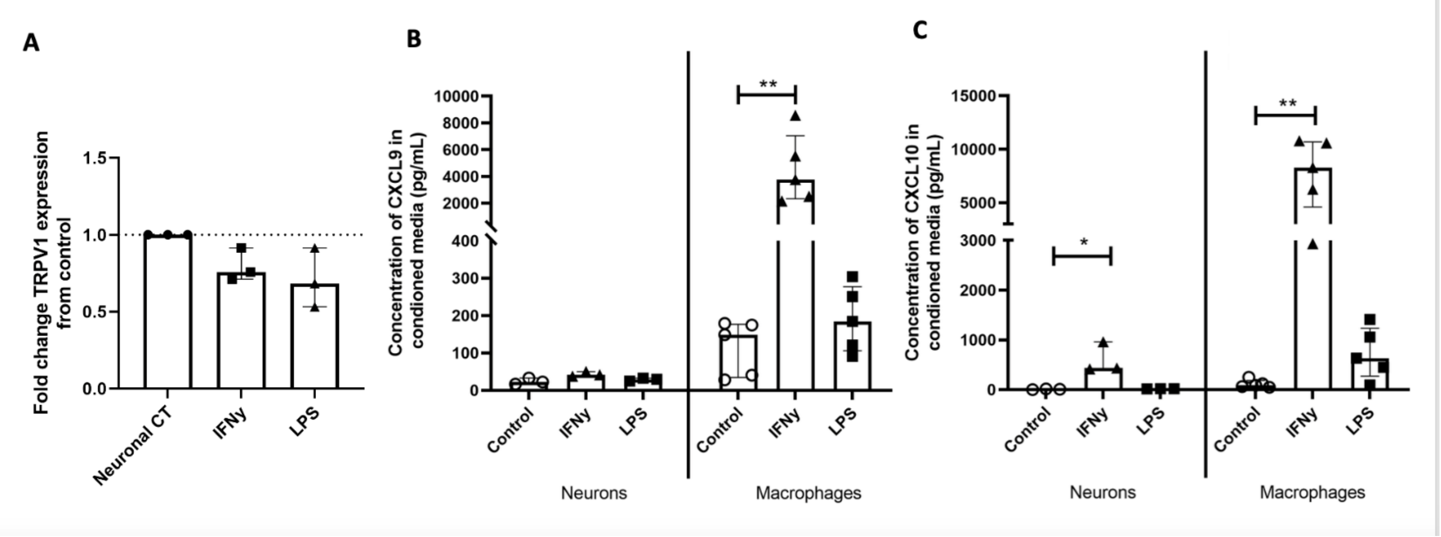

Figure S2. iPSC-PSNs did not upregulate expression of TRPV1 and released low amounts of CXCL9 and CXCL10 following treatment with IFNg or LPS. (A) Fold change TRPV1 gene expression of iPSC-PSNs (n=3 separate differentiations) treated with IFN**g** 10 ng/mL or LPS 10 ng/mL for 24 hrs. Dotted line is the comparison to control condition. (B) Concentration of CXCL9 (pg/mL) in conditioned media from neurons (KW: P=0.05) or MDMs (KW: P=0.0006) treated with IFN**g** 10 ng/mL or LPS 10 ng/mL for 24 hrs. (C) Concentration of CXCL10 (pg/mL) in conditioned media from neurons (KW: P=0.004) or MDMs (KW: P<0.0001) treated with IFN**g** 10 ng/mL or LPS 10 ng/mL for 24 hrs. Statistical analysis was performed using a Kruskal-Wallis one-way analysis of variance and Dunn's multiple comparison test. *p< 0.05, **p< 0.01. Error bars indicate the median and IQR. .

Table S1. Viral and Pathological Information of Animals Used in RNAseq.

	Animal ID	Sex	VL (Log10 copies/mL) *	Age (Years)	Days post infection (DPI) survival	DRG pathology score**
SIV-	A01	Male	N/A	10.8	N/A	N/A
	A02	Male	N/A	6.7	N/A	N/A
	A03	Female	N/A	7.8	N/A	N/A
	A04	Male	N/A	6.7	N/A	N/A
SIV+	A05	Male	7.79	5.4	97	1
	A06	Male	8.54	5.8	77	2
	A07	Male	7.67	11.5	146	2.5
	A08	Male	7.83	7.3	55	1.5
	A09	Male	5.41	6.3	84	3
SIV-+/AR	A10	Male	2.87	10.4	120	1

	A11	Male	2.34	6.2	120	1
	A12	Male	2.66	6.2	118	1
	A13	Male	2.66	6.1	118	1
	A14	Male	3.62	10.3	120	1

* Plasma viral load at time of necropsy.

** General scoring includes lumbar and sacral DRG for pathology score. Pathologic grading and criteria based on evaluation from a certified veterinary pathologist as previously reported.
